# Antagonistic effects of selenium on cadmium-induced apoptosis by restoring the mitochondrial dynamic equilibrium and energy metabolism in chicken spleens

**DOI:** 10.18632/oncotarget.17539

**Published:** 2017-04-30

**Authors:** Zhe Xu, Xi Jin, Tingru Pan, Tianqi Liu, Na Wan, Shu Li

**Affiliations:** ^1^ College of Veterinary Medicine, Northeast Agricultural University, Harbin, 150030, P. R. China

**Keywords:** cadmium, selenium, spleens, mitochondrial dynamics, apoptosis

## Abstract

The aim of this study was to investigate the mechanism of cadmium-induced apoptosis in chicken spleens and the antagonistic effects of selenium. We duplicated the selenium-cadmium interaction model and examined the expression of apoptosis-, immune-, mitochondrial dynamics- and energy metabolism-related genes. The results demonstrated that after treatment with cadmium, the frequency of apoptosis was significantly increased, and the morphological characteristics of apoptosis were observed. The expression of pro-apoptotic genes was increased, and that of anti-apoptotic genes was decreased. The mRNA levels of tumor necrosis factor-α and interlenkin-1β were observably increased, but the interlenkin-2 and interferon-γ levels were markedly decreased. Furthermore, the mRNA and protein levels of dynamin-related protein 1 and mitochondrial fission factor were significantly enhanced, whereas mitofusin 1, mitofusin 2, and optic atrophy 1 were markedly decreased. The expression of hexokinase 1, hexokinase 2, aconitase 2, lactate dehydrogenase A, lactate dehydrogenase B, succinatedehydrogenase B, pyruvate kinase and phosphofructokinase were also reduced. Selenium supplements remarkably attenuated cadmium-induced effects (*p* < 0.05). Based on the above results, conclude that the cadmium treatment promoted a mitochondrial dynamic imbalance and reduced energy metabolism, leading to apoptosis and immune dysfunction in chicken spleens, and selenium had an antagonistic effect on Cd-induced apoptosis.

## INTRODUCTION

Cadmium (Cd) is a widespread heavy metal pollutant, which can be discharged into the environment during industrial production, including the production of batteries, metal plating, pigments and plastics [1]. Heavy metals may be ingested by animals via respiration or gastrointestinal absorption, resulting in the possible risk of harm to humans through the food web [2, 3]. Cd accumulates in various tissues and cells and produces toxic effects, such as immune dysfunction and apoptosis. In caprine spleens, the combination of Cd and molybdenum induced noteworthy damage by promoting cell apoptosis [4]. In the immune organs of chicken, arsenic trioxide exposure increased the levels of heat shock protein, disrupting immune function [5]. Cd was found to induce oxidative stress, endoplasmic reticulum stress, and NO overproduction, which are factors that are believed to be involved in apoptosis [6, 7]. Several studies have reported that Cd induces apoptosis through a mitochondria-dependent pathway. In rat proximal tubular cells, Cd induced apoptosis by the breakdown of mitochondrial ΔΨ and overproduction of reactive oxygen species (ROS) [8, 9]. The breakdown of mitochondrial ΔΨ and the increased mitochondrial membrane permeability resulted in the release of cytochrome c (Cyt-c) from the mitochondria into the cytosol [10]. Furthermore, Cd increased expression of pro-apoptosis genes (such as Bax and Bak) and decreased expression of anti-apoptosis genes (such as Bcl-2), which may induce the release of Cyt-c [11]. In pancreatic β-cells, Cd increased the expression of p53, leading to the release of Cyt-c and apoptosis [12]. Cyt-c activated the caspase protein family (such as caspase 3 and caspase 9) and led to apoptosis [13]. Recent studies have suggested that Cd induces mitochondrial fragmentation by increasing dynamin-related protein 1 (Drp1) expression [14, 15]. Manganese (Mn) also induced deregulation of expression levels of mitochondria-shaping proteins and exacerbated fragmentation [16]. Mitochondrial fragmentation, in turn, facilitated apoptosis [17]. The Drp1 and mitochondrial fission factor (Mff) mediated mitochondrial membrane fission. Conversely, mitofusin 1 (Mfn1), mitofusin 2 (Mfn2) and optic atrophy 1 (Opa1) mediated mitochondrial membrane fusion. Additionally, several reports revealed that heavy metal toxicity led to energy metabolism dysfunction, resulting in apoptosis. In skeletal muscles of mice, Cd inhibited the expression of hexokinase (HK) and phosphofructokinase (PFK), disrupting glycolysis and decreasing ATP generation [18]. The reduced glucose metabolism and energy production impaired the normal structure and function of cardiomyocytes, leading to apoptosis [19].

Selenium (Se) is an essential trace mineral for animals and humans [20, 21]. Se has antioxidant effects [22] and improves the body's immune system [23]. Se deficiency causes various diseases, such as hepatocellular carcinoma and muscular dystrophy [24–27]. Many studies have indicated that Se can alleviate toxicity induced by several heavy metals and drugs [28]. Se antagonized lead (Pb)-induced over-expression of inflammatory cytokines in the peripheral blood lymphocytes of chickens [29]. In chicken splenic lymphocytes, Se partly attenuated Cd-induced immune toxicity by improving the expression of immune cytokines [30]. More recently, several reports have indicated that Se has protective effects against Cd-induced apoptosis. In mice kidneys, Se markedly inhibited apoptosis attributed to Cd-induced mitochondrial dysfunction [30]. The alleviating effects of Se on Cd-induced apoptosis involved downregulating the expression of pro-apoptotic genes (Bak, p53, caspase 3 and caspase 9) and upregulating the expression of anti-apoptotic genes (Bcl-2 and Bcl-x) [13, 31]. Moreover, Khera et al. suggested that Se supplementation increased the mitochondrial contents and increased the expression of mediators of mitochondrial biogenesis in cells [32]. Se was also found to alleviate Cd-induced changes in tissues and mitochondria ultrastructure [33].

In summary, several previous studies have demonstrated that Cd induces apoptosis in various tissues and cells, and Se can relieve Cd toxicity. Nevertheless, the mechanisms underlying Cd-induced apoptosis by disrupting mitochondrial dynamics and energy metabolism in chicken spleens and the antagonistic effects of Se are still unclear. The aim of this study was to investigate these mechanisms. We established the Se-Cd interaction model and examined the ultrastructural changes, frequency of apoptosis and expression of apoptosis-, immune-, mitochondrial dynamic- and energy metabolism-related genes in chicken spleens. With this study, we hope to provide a new perspective on the interaction of Se and Cd-induced apoptosis in chicken spleens.

## RESULTS

### Ultrastructural changes

**Figure 1:**
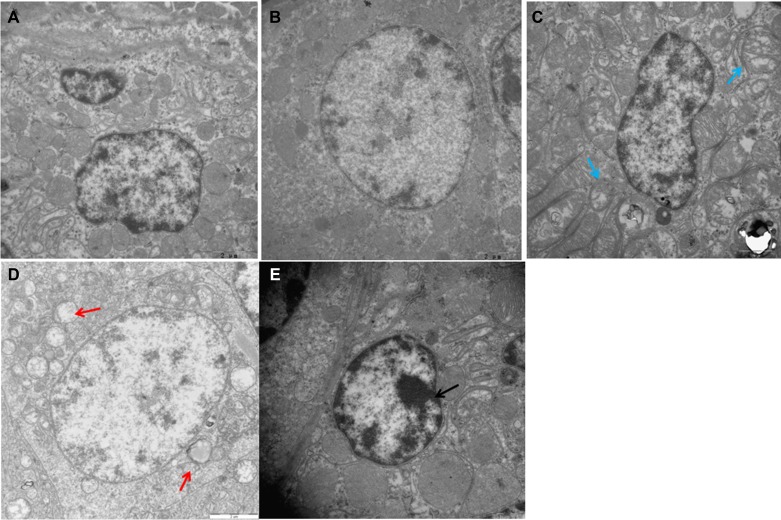
Ultrastructural changes in chicken spleens (**A**) Spleens of the control group (×15000). (**B**) Spleens of the Se-treated group (×15000). (**C**) Spleens of the Se+Cd-treated group (×15000). (**D**) Spleens of the Cd-treated group (×15000). (**E**) Spleens of the Cd-treated group (×20000). Blue arrow displays slightly swollen mitochondria. Red arrow displays mitochondrial vacuolation. Black arrow displays chromatin condensation.

Electron microscopy showed normal ultrastructure of chicken spleens in the control and Se-treated groups (Figure 1A–1B), with no obvious pathological changes. As shown in Figure 1D–1E, Cd treatment alone caused extensive splenic tissue damage. The apoptotic cells displayed morphological characteristics of apoptosis, including cell shrinkage, chromatin condensation, and margination. Furthermore, Cd treatment caused distinctly swollen mitochondria with degenerating or missing cristae. In the Se+Cd-treated group, slightly dilated cisternae of the smooth endoplasmic reticulum and swollen mitochondria were observed (Figure 1C).

### TdT-mediated dUTP nick end labeling (TUNEL) assay

**Figure 2:**
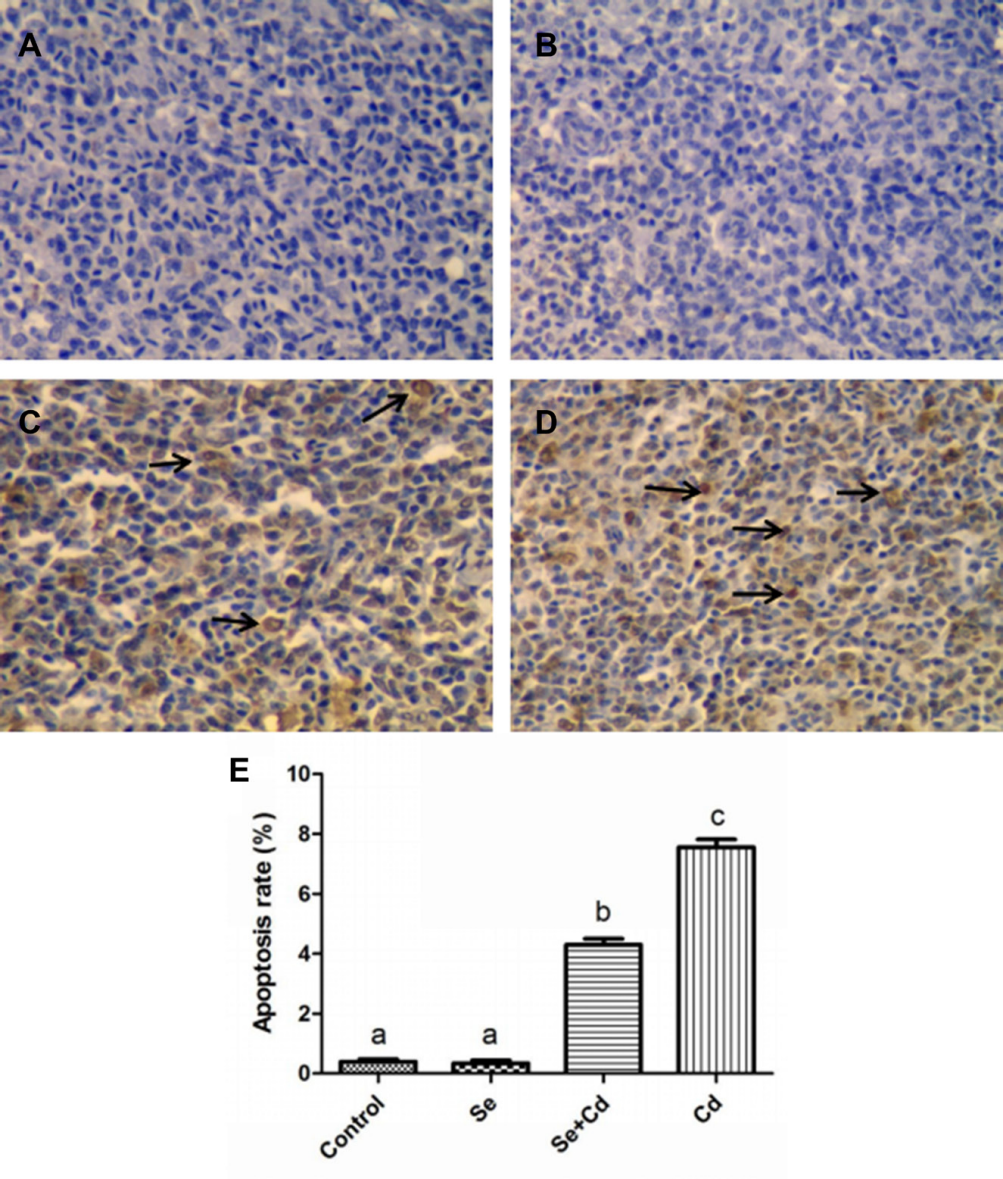
The frequency of apoptosis in chicken spleens detected by TUNEL assay TUNEL staining (**A**–**D**): spleens of the control group, the Se-treated group, the Se+Cd-treated group, and the Cd-treated group. The magnification is ×400 and the black arrow displays the apoptotic cells. (**E**) The percentage of the apoptotic cells. Each value represents the mean ± SD. Bars not sharing a common letter are significantly different (*p* < 0.05).

The number of apoptotic cells in chicken spleens was examined by the TUNEL assay. The apoptotic cells had brown-stained nuclei, and showed morphological changes such as condensed and irregular nuclei. As is presented in Figure 2A–2B, the number of apoptotic cells in spleens was similar between the control group and the Se-treated group. In the Cd-treated group, the number of apoptotic cells was evidently increased compared to the other groups (Figure 2C). Se supplementation during Cd exposure markedly reduced the frequency of apoptotic cells compared to the Cd-treated group (Figure 2D).

### The relative expression of apoptosis-related genes in chicken spleens

**Figure 3:**
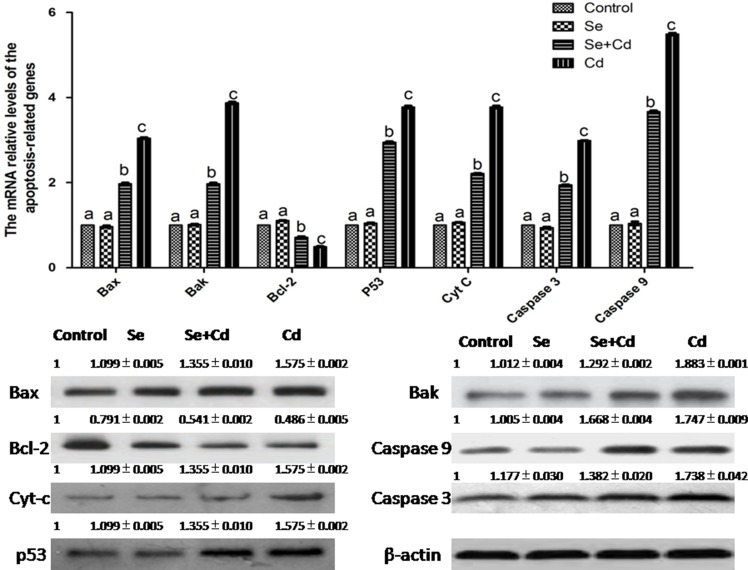
The relative mRNA and protein levels of apoptosis-related genes in chicken spleens Effects of Se, Cd and Se+Cd treatment on the relative mRNA and protein levels of apoptosis-related genes were examined in chicken spleens. Chicken β-actin was used as an internal reference. Each value represents the mean ± SD (*n* = 10). Bars with different small letters represent statistically significant differences between the groups (*p* < 0.05); the bars with a common letter are not significantly different (*p* > 0.05).

The expression of apoptosis-related genes in chicken spleens is shown in Figure 3. Se treatment alone had no influence on apoptosis-related genes compared to those in the control group (*p* > 0.05). In the Cd-treated group, mRNA and protein levels of Bax, Bak, p53, Cyt-c, caspase 3 and caspase 9 were markedly increased and those of Bcl-2 were significantly decreased compared to the levels in the control group (*p* < 0.05). In the Se+Cd-treated group, we found that Se observably attenuated the changes induced by Cd, but expression levels did not reach the control group levels (*p* < 0.05).

### The relative expression of immune-related genes in chicken spleens

**Figure 4:**
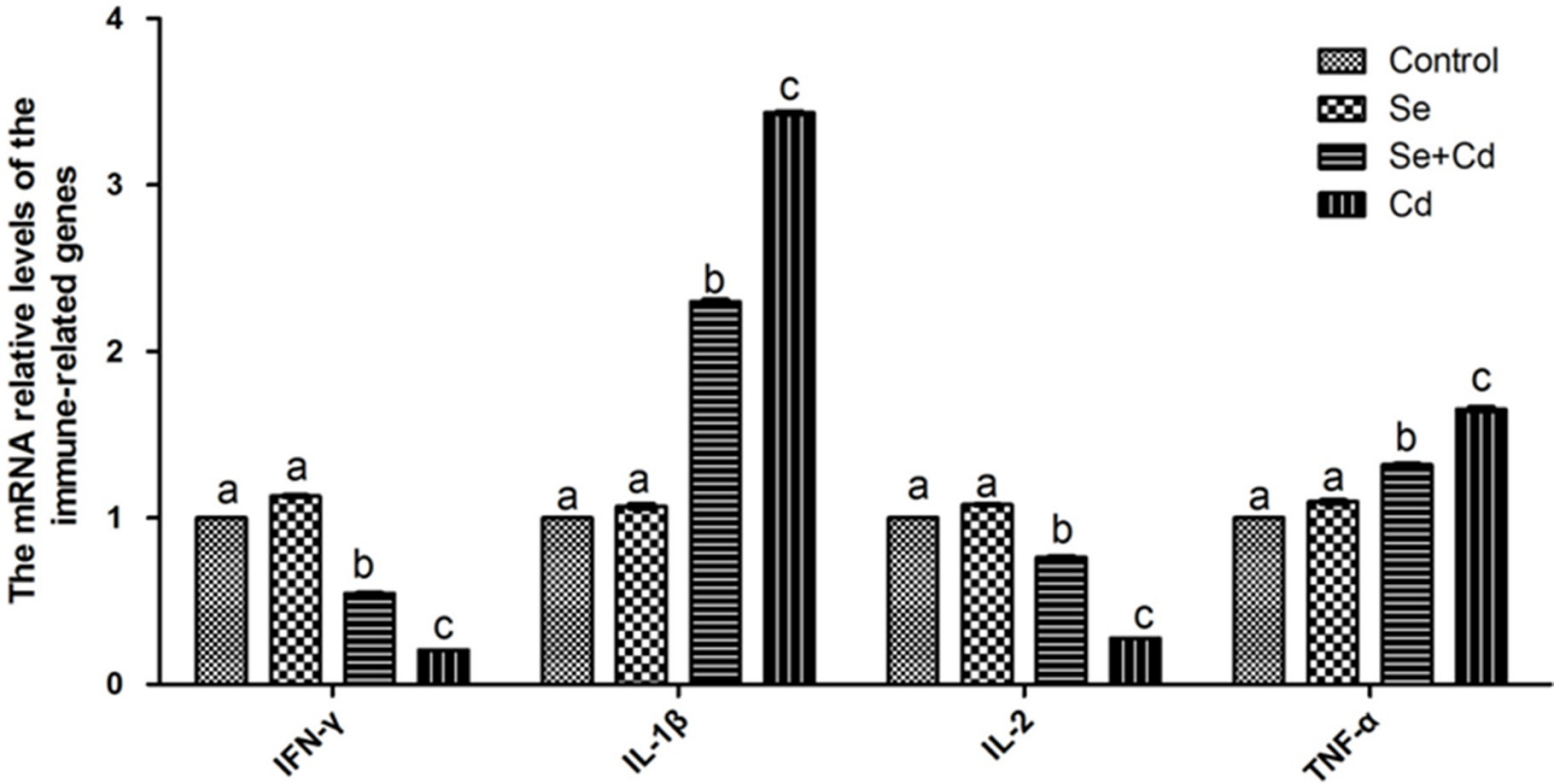
The relative mRNA levels of immune-related genes in chicken spleens Effects of Se, Cd and Se-Cd treatment on the relative mRNA and protein levels of immune-related genes were examined in chicken spleens. Chicken β-actin was used as an internal reference. Each value represents the mean ± SD (*n* = 10). The bars with different small letters represent statistically significant differences between the groups (*p* < 0.05); the bars with a common letter are not significantly different (*p* > 0.05).

The expression of immune-related genes in chicken spleens is shown in Figure 4. There were no significant differences between the expression levels in the control group and the Se-treated group (*p* > 0.05). The relative mRNA levels of interlenkin-1β (IL-1β) and tumor necrosis factor-α (TNF-α) were markedly higher, and interlenkin-2 (IL-2), interferon-γ (IFN-γ) levels were remarkably lower in the Cd-treated group than those in the control group (*p* < 0.05). Se and Cd co-treatment significantly attenuated the changes induced by Cd, but did not bring the gene expression to the control group level (*p* < 0.05).

### The relative expression of mitochondrial dynamics-related genes in chicken spleens

**Figure 5:**
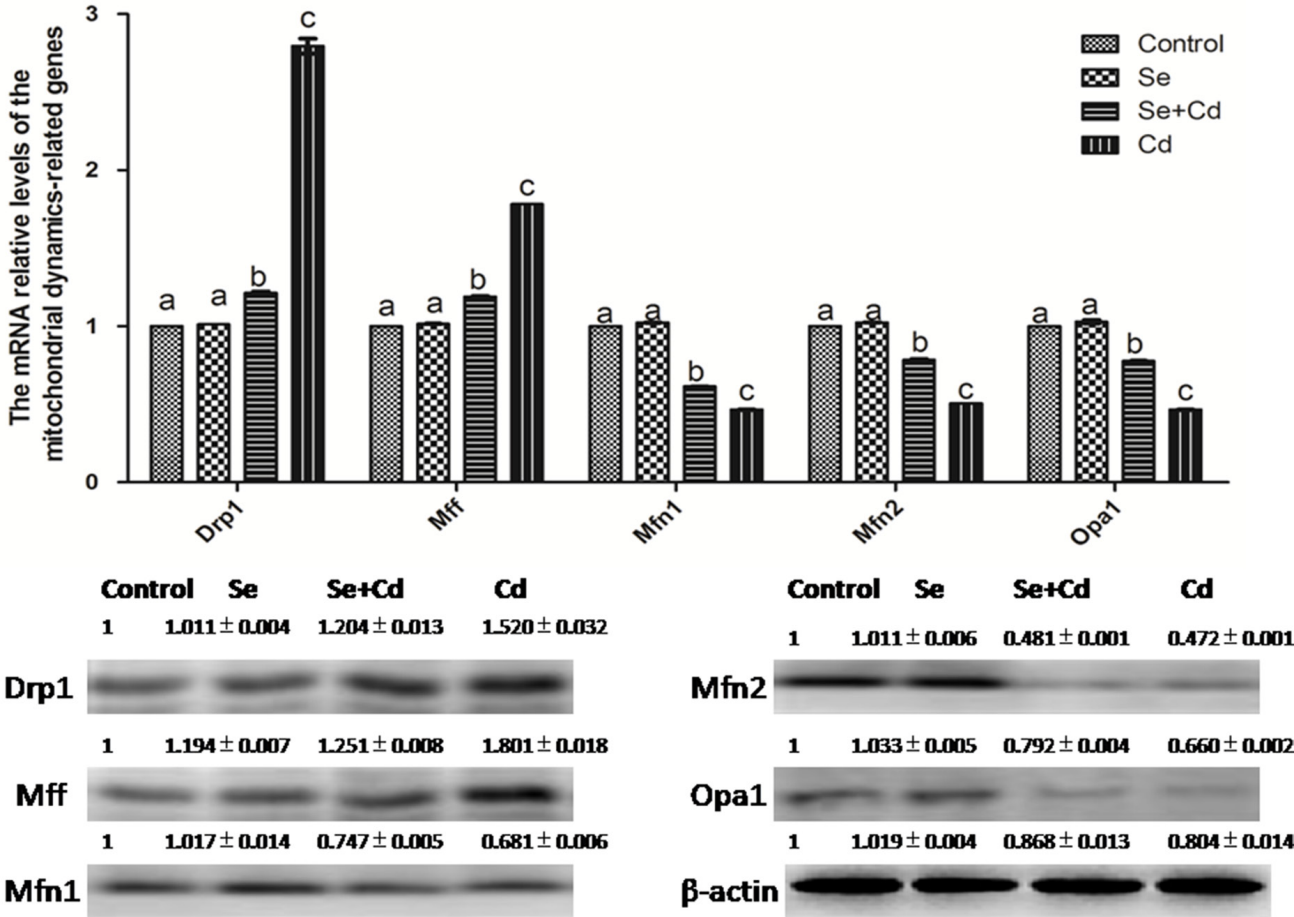
The relative mRNA and protein levels of mitochondrial dynamics-related genes in chicken spleens Effects of Se, Cd and Se+Cd treatment on the relative mRNA and protein levels of mitochondrial dynamics-related genes were examined in chicken spleens. Chicken β-actin was used as an internal reference. Each value represents the mean ± SD (*n* = 10). The bars with different small letters represent statistically significant differences between the groups (*p* < 0.05); the bars with a common letter are not significantly different (*p* > 0.05).

The effects of Cd and/or Se on the relative expression of mitochondrial dynamics-related genes in chicken spleens are shown in Figure 5. Se treatment alone did not influence the mitochondrial dynamics-related genes compared to the control group genes (*p* > 0.05). Cd treatment alone significantly decreased the mRNA and protein levels of Mfn1, Mfn2 and Opa1, and elevated the mRNA and protein levels of Drp1 and Mff, compared to the control group levels (*p* < 0.05). However, in the Se+Cd-treated group, we observed that Se remarkably decreased the mRNA and protein levels of Mff and Drp1, and increased the mRNA and protein levels of Opa1, Mfn2 and Mfn1 compared to the levels in the Cd-treated group (*p* < 0.05).

### The relative expression of energy metabolism-related genes in chicken spleens

**Figure 6:**
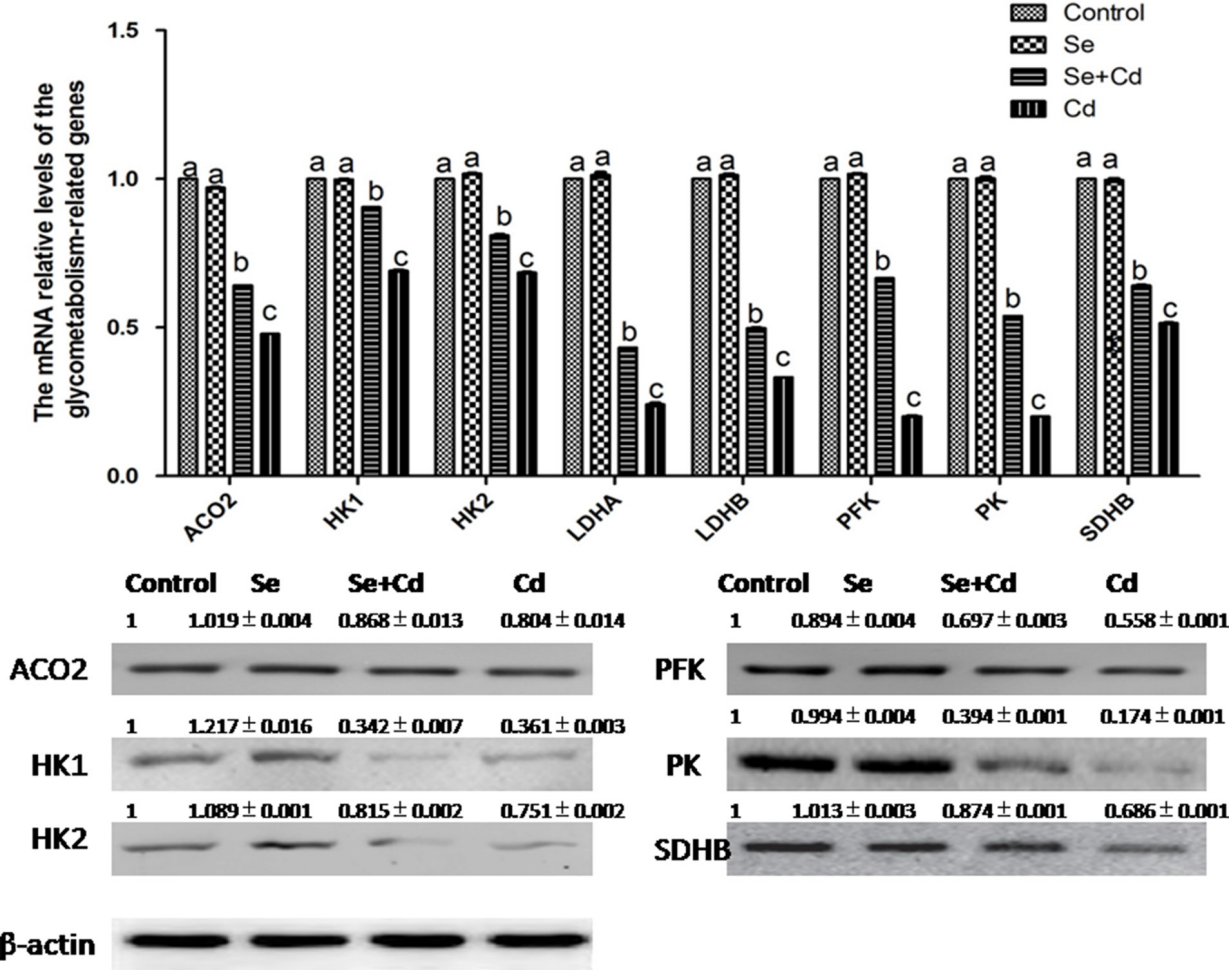
The relative mRNA and protein levels of energy metabolism-related genes in chicken spleens Effects of Se, Cd and Se+Cd treatment on the relative mRNA and protein levels of energy metabolism-related genes were examined in chicken spleens. Chicken β-actin was used as an internal reference. Each value represents the mean ± SD (*n* = 10). The bars with different small letters represent statistically significant differences between the groups (*p* < 0.05); the bars with a common letter are not significantly different (*p* > 0.05).

The relative expression of energy metabolism-related genes in chicken spleens is shown in Figure 6. Se treatment alone did not influence the energy metabolism-related genes compared to the control group genes (*p* > 0.05). Cd treatment alone remarkably decreased the expression of aconitase 2 (ACO2), hexokinase 1 (HK1), hexokinase 2 (HK2), lactate dehydrogenase A (LDHA), lactate dehydrogenase B (LDHB), PFK, pyruvate kinase (PK) and succinatedehydrogenase B (SDHB) compared to the expression levels in the other groups (*p* < 0.05). However, in the Se+Cd-treated group, the expression of ACO2, HK1, HK2, LDHA, LDHB, PFK, PK and SDHB was markedly increased compared to the Cd-treated group expression levels (*p* < 0.05).

## DISCUSSION

Apoptosis plays an important role in stabilizing the homeostatic system in organisms [34]. Because the spleen is a primary immune organ, excessive apoptosis in splenic cells may cause structural and functional damage to the spleen, leading to immune dysfunction. Many researchers have observed that Cd induces apoptosis in animals. For instance, in rats, Cd treatment increased the number of apoptotic cells in renal cortical tissues and brain [35, 36]. In chicken, Cd induced apoptosis in the liver, leading to liver tissue damage [37]. In the present study, our results demonstrated that splenic cells with Cd treatment displayed typical morphological changes associated with apoptosis, and the frequency of apoptosis was markedly increased. However, Se was reported to have antagonistic effects on Cd toxicity in the immune organs of chicken [38]. Consistent with these studies, our results suggested that Se may significantly recover the normal structure of splenic cells, reduce the frequency of Cd-induced apoptotic cells, restore spleen function and enhance immune function.

Several studies have demonstrated that Cd has an immunosuppressive effect. IL-1β, IL-2, TNF-α and IFN-γ are important indexes of immune function [39–41]. Pb exposure increased the level of TNF-α [42, 43]. Excessive Mn inhibited the expression of IL-2, causing immunosuppression in chicken splenic lymphocytes [43]. Our results demonstrated that the mRNA and protein levels of TNF-α and IL-1β were markedly increased and those of IL-2, IFN-γ were significantly decreased following Cd treatment. These results support findings from previous studies and confirm that Cd induces immune dysfunction in chicken spleen. In addition, reports demonstrated that Pb toxicity was remarkably alleviated by Se treatment in the chicken liver [44]. Consistent with these reports, our results also demonstrated that Se supplements could rescue the abnormal expression of immune-related genes induced by Cd, thereby improving immune function.

A recent study demonstrated that mitochondrial dynamic imbalances induced apoptosis [45]. Drp1, Mff, Mfn1, Mfn2 and Opa1 are associated with the mitochondrial dynamics equilibrium, and Drp1 and Mff play a major role in mitochondrial fission. Conversely, mitochondrial fusion is largely regulated by Mfn1, Mfn2 and Opa1. In addition, Drp1-dependent cristae remodeling amplify apoptosis [45]. Opa1 not only promotes mitochondrial fusion [46] but also regulates the cristae shape and remodeling to control Cyt-c release [47]. Studies have demonstrated that CdCl_2_ exposure increased Drp1 expression and enhanced mitochondrial recruitment, resulting in excessive mitochondrial fission [14]. The excessive mitochondrial fission led to mitochondrial fragmentation and mitochondrial structure damage, causing Cyt-c release [48]. Cyt-c, in turn, activated the caspase cascade, leading to apoptosis. Moreover, Drp1 promoted Bax mitochondrial translocation [49]. Bax and Bak also inhibited mitochondrial fusion by impinging on Mfn2 [50]. Bax and BcL-2 increased mitochondrial permeability and induced apoptosis. Samy et al. observed that the expression of Bax and Bak was markedly increased and that of Bcl-2 was decreased by Cd [51]. In addition, treatment with Cd increased the expression of p53 and caused apoptosis [52]. Consistent with the results of previous studies, we found that the expression of Drp1 and Mff was markedly enhanced and expression of Mfn1, Mfn2 and Opa1 was decreased in chicken spleens following Cd treatment. Evidently swollen mitochondria with degenerating or missing cristae were observed, and the expression of caspase 3, caspase 9 and p53 was markedly increased in the Cd-treated group. Previous studies revealed that Se supplements restored the normal structure of splenic cells [38] and prevented mitochondrial dynamic imbalance [53]. Our results are consistent with these results and demonstrate that Se suppressed expression of Drp1 and Mff and enhanced expression of Mfn1, Mfn2 and Opa1, consequently restoring mitochondrial dynamics equilibrium in chicken spleens. Furthermore, as described in a study by Liu [13], our results also demonstrated that Se markedly reduced Cd-induced apoptosis in chicken spleens by decreasing the expression of Bax and Bak and increasing the expression of Bcl-2.

Mitochondria are the primary sites for production of ATP. If mitochondrial function was impaired, the energy metabolism system would be in disorder. Because glycometabolism is the primary method to produce energy, many reports have investigated whether cell apoptosis via the mitochondrial pathway may be related to glycometabolism. In rats, ATP reduction induced by inhibiting glycometabolism triggered cell apoptosis [54]. In Panc-1 human pancreatic cancer cells, the apoptotic rate gradually increased due to blocked glycometabolism [55]. Evidence from previous studies suggests that HK2 depression inhibits human and mouse lung cancer cell growth by inducing cell apoptosis [56]. LDH inhibition also promoted apoptosis through enhancement of mitochondrial oxidative stress generation [57]. In our study, we detected the expression of a series of glycometabolism-related enzymes, including LDHA, LDHB, HK1, HK2, PK, SDHB, PFK and ACO2. Consistent with previous findings, our results showed that the levels of all the glycometabolism-related enzymes detected were decreased with Cd treatment. Therefore, Cd inhibited both aerobic and anaerobic respiration by reducing the expression of glycometabolism-related genes, resulting in apoptosis in chicken spleens. Moreover, Lu reported that sodium selenite improved glycometabolism [58]. Our results are in support of previous research findings and confirm that Se improves glycometabolism by increasing the expression of LDHA, LDHB, HK1, HK2, PK, SDHB, PFK and ACO2 in chicken spleens.

**Figure 7:**
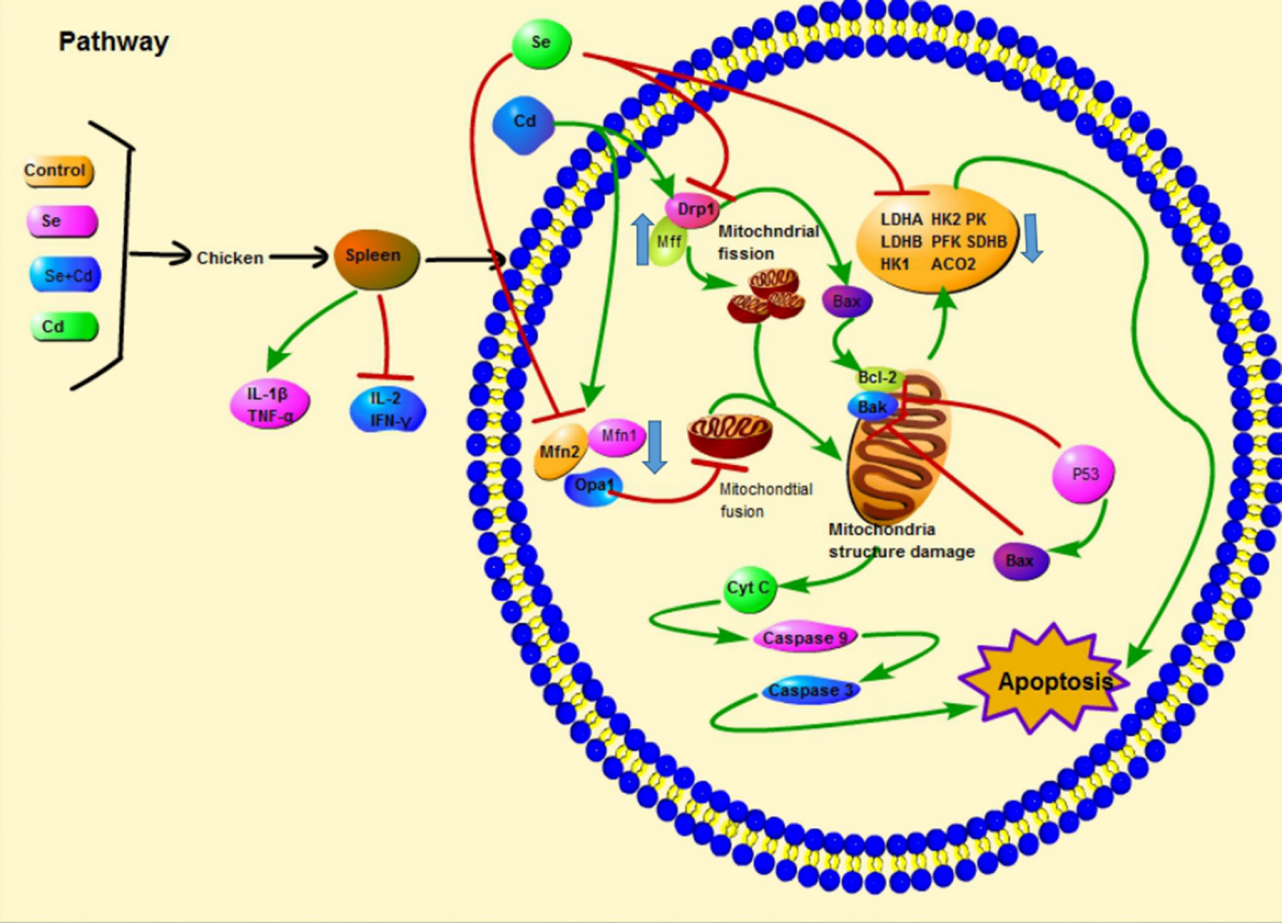
Scheme shows the pathway of Se and Cd interaction and the Cd-induced apoptosis in chicken spleens Scheme shows the mechanism of Cd-induced apoptosis by disturbing mitochondrial dynamics and energy metabolism in chicken spleens and the antagonistic effects of Se. The green line displays the promotion and the red line displays the inhibition.

In conclusion, we demonstrated that Cd induced excessive mitochondrial fragmentation, mitochondrial structure damage and reduced energy metabolism, leading to apoptosis and immune dysfunction in chicken spleens, and Se inhibited apoptosis by partially alleviating (Figure 7) these effects induced by Cd. Our results enrich the understanding of the mechanism of apoptosis induced by Se and Cd interaction in chicken spleens.

## MATERIALS AND METHODS

### Birds and experimental design

The experiments were approved by the Institutional Animal Care and Use Committee of the Northeast Agricultural University under the approved protocol number SRM-06. We determined the Cd lethal dose (LD50) for chicken to be 218.44 mg/kg BW, and the doses and durations of Cd and Se used in this study have been described previously [59]. In brief, forty-eight 28-day-old Isa Brown male chickens were divided randomly into four groups (*n* = 10 per group). The remaining two chickens in each group were used as standby in case of any unexpected conditions. Group I (the control group) was fed a basal diet without Se or Cd supplementation (the Se content was 0.2 mg/kg). Group II (the Se-treated group) was fed the basic diet supplemented with Na_2_SeO_3_; the total Se content was 2 mg/kg. Group III (the Se+Cd-treated group) was fed the basic diet supplemented with Na_2_SeO_3_ (the total Se content was 2 mg/kg) and 150 mg/kg CdCl_2_. Group IV (the Cd-treated group) was fed the basic diet supplemented with 150 mg/kg CdCl_2_. The duration of experiment was 90 days and the 10 chickens in each group were selected randomly for detection of experimental indexes. Following euthanasia, part of the spleen was examined by electron microscopy and the TUNEL assay, as described in the following sections. The remaining spleen was quickly removed and cleaned with ice-cold sterile deionized water, frozen immediately in liquid nitrogen, and stored at −80°C until required for subsequent experiments.

### Transmission electron microscopy

For electron microscopy, spleen tissue specimens (approximately 1 mm^3^) were rapidly fixed with 2.5 % glutaraldehyde in 0.1 M sodium phosphate buffer (pH 7.2) for 3 h at 4°C, washed in the same buffer for 1 h at 4°C, and post-fixed with 1% osmium tetroxide in sodium phosphate buffer for 1 h at 4°C. The tissues were then dehydrated in a graded series of ethanol, starting at 50 % for 10 min at each step, followed by two changes in propylene oxide. The tissue specimens were embedded in Araldite. Ultrathin sections were stained with Mg-uranyl acetate and lead citrate for transmission electron microscope evaluation (GEM-1200ES, Japan).

### TUNEL assay

TUNEL detection of apoptotic cells was performed in formalin-fixed, paraffin-embedded spleen tissue sections according to the manufacturer's protocol (Roche, USA). This procedure distinguishes apoptotic cells from those undergoing necrosis because damaged DNA in apoptotic cells leads to a different distribution of staining and nuclear morphology. Paraffin wax-embedded tissue sections were treated with proteinase K, and the endogenous peroxidase activity was blocked with hydrogen peroxide. The sections were incubated at 37°C with the terminal TdT/nucleotide mixture for 1 h. Next, the reaction was stopped and the slides were rinsed with phosphate-buffered saline (PBS). Nuclear labeling was developed with horseradish peroxidase and diaminobenzidine. Hematoxylin was used for counterstaining.

### Quantitative real time-polymerase chain reaction

Total RNA was isolated from tissue samples using the TRIzol reagent according to the manufacturer's instructions (Invitrogen, Carlsbad, USA). The RNA concentrations were determined using the GeneQuant 1300. The procedure for reverse transcription was performed according to the manufacturer's instructions (Roche, Basel, Switzerland). Synthesized cDNA was stored at −20°C for PCR. Oligo 6.0 software was used to design specific primers based on known sequences (Table 1). Chicken β-actin was used as a housekeeping gene and an internal reference. Primers were synthesized by Invitrogen Biotechnology Co. Ltd. in Shanghai, China.

**Table 1:** Gene-specific primers used in the real-time quantitative reverse transcription PCR experiments

Gene	Primer (5′-3′)	Gene	Primer (5′-3′)
Mff	F:TGGGAAGGCTGAAGAGAGAA	Drp1	F:GGCAGTCACAGCAGCTAACA
	R:GGTGTTCCCTCAAGTGTGGT		R:GCATCCATGAGATCCAGCTT
Opa1	F:GCTACGGACCAGGGTTATGA	Mfn1	F:TGAGCATGTAGCAACGGAAG
	R:GCTCAAGCATCCGTTGGTAT		R:AGCAAGCTGATTGACGGTCT
Mfn2	F:TACCAGGCAGATTTCCATCC	IFN-γ	F:AGCCGCACATCAAACACATA
	R:GTGATTGCATTGGAACAACG		R:CGCTGGATTCTCAAGTCGTT
TNF-α	F:AGATGGGAAGGGAATGAACC	IL-1β	F:CTCCTCCAGCCAGAAAGTGA
	R:ACTGGGCGGTCATAGAACAG		R:GAGCTTGTAGCCCTTGATGC
IL-2	F:TGCAGTGTTACCTGGGAGAA	β-actin	F:ACGTCGCACTGGATTTCGAG
	R:CGGTGTGATTTAGACCCGTAA		R:TGTCAGCAATGCCAGGGTAC
PK	F:GAACTGCGATGAGAATGTGC	HK2	F:CCCAGATAGAAAGCGACTGC
	R:ACCAGCAAGGAAATGAGACC		R:ACCTCCTTGACGATGATGCT
SDHB	F:GCTGCGGCCGATCTGT	PFK	F:GAGCCACCTGAACATCGTG
	R:GCTTGTCCCCAGGCTTATCA		R:CATCACTTCCAGCACAAACG
HK1	F:ACTTCACCAAACGAGGGTTC	LDHA	F:CATCACTTCCAGCACAAACG
	R:CCTGTCGCTCTCAATCTGTG		R:CGGTGTTTAGGAAAGCCACT
LDHB	F:AAGCAGGTTGTTGAAAGTGC	ACO2	F:GCCAAGGACATAAACCAGGA
	R:AAGGCAGGCTCAGGAAGAC		R:TGTGAGTCTGTGCCAATCAAC
Bax	F:TATGGGACACCAGGAGGGTA	Bak	F: ACCCGGAGATCATGGAGA
	R:CGTAGACCTTGCGGATAAAGC		R:GATGCCTTGCTGGTAGACG
P53	F:CCCATCCTCACCATCCTTACA	Bcl-2	F:ATCGTCGCCTTCTTCGAGTT
	R:CTCGATCTTGCGGTCCCTC		R:CTGACTATCACCAAGAACCACC
Caspase 3	F:CTGAAGGCTCCTGGTTTA	Caspase 9	F:CCGAAGGAGCAAGCACG
	R:CTCGATCTTGCGGTCCCTC		R:AGGTTGGACTGGGATGGAC
Cty-c	F:AGGCAAGCACAAGACTGGA		
	R:CTGACTATCACCAAGAACCACC		

QRT-PCR was performed on a LightCycler^®^480 Detection System (Roche, Basel, Switzerland) using Fast Universal SYBR Green Master (Roche, Basel, Switzerland). The program was run as follows: 1 cycle at 95°C for 30 s followed by 40 cycles at 95°C for 5 s and at 60°C for 34 s. Dissociation curves for each PCR reaction were analyzed by using the Dissociation Curve 1.0 software (Applied Biosystems) to detect and eliminate possible primer-dimers and non-specific amplifications. The relative abundance of mRNA was calculated according to the method of Pfaffl [60].

### Western blot analysis

Equal amounts of total protein (40 μg) were subjected to 12 % SDS-polyacrylamide gel electrophoresis under reducing conditions. The separated proteins were then transferred to nitrocellulose membranes using a tank transfer for 2h at 200mA in Tris-glycine buffer containing 20 % methanol. Membranes were blocked with 5 % skim milk for 24 h and incubated overnight with diluted primary antibodies against Bcl-2, Bak, Bax, Cyt-c (1:500, Santa Cruz Biotechnology, USA), Mff, Drp1, Mfn1, Mfn2, Opa1 (1:500, Proteintech, Chicago, IL, USA), caspase 3, caspase 9, p53, PK, HK1, HK2, AOC2, SDHB and PFK (1:1000, Santa Cruz Biotechnology, CA USA). Bound primary antibodies were detected with a horseradish peroxidase (HRP) conjugated secondary antibody against rabbit IgG (1:1500, Santa Cruz, CA, USA). To verify equal loading of the samples, the membrane was incubated with a monoclonal β-actin antibody (1:1000, Santa Cruz, CA, USA), followed by incubation with an HRP conjugated goat anti-mouse IgG (1:1000). The signal was measured by enhanced chemiluminescence detection reagents (Applygen Technologies Inc., Beijing, China). Protein bands were visualized using a ChampChemi imaging system (Beijing Sage Creation Science Co. Ltd., Beijing, China). The relative abundances of the proteins were expressed as the ratios of optical density of each of these proteins to that of β-actin.

### Statistical analysis

Statistical analyses of all data were performed using SPSS for Windows (version 21.0; SPSS Inc., Chicago, IL, USA). One-way analysis of variance followed by the Tukey's honest significant difference test was used to analyze the descriptive statistics (mean values, standard deviation). The results were considered to be significant when *p* < 0.05.

## Abbreviations

Se: selenium
Cd: cadmium
BW: body weight
HK1: hexokinase 1
HK2: hexokinase 2
LDHA: lactate dehydrogenase A
LDHB: lactate dehydrogenase B
ACO2: aconitase 2
PK: pyruvate kinase
PFK: phosphofructokinase
SDHB: succinatedehydrogenase B
IL-1β: interlenkin-1β
IL-2: interlenkin-2
TNF-α: tumor necrosis factor-α
IFN-γ: interferon-γ
MFF: mitochondrial fission factor
Drp1: dynamin-related protein 1
Opa1: optic atrophy 1
Mfn1: mitofusin 1
Mfn2: mitofusin 2
Cyt-c: cytochrome c
ROS: reactive oxygen species
Mn: manganese
Pb: lead
